# Association of serum cotinine with phenotypic age acceleration and oxidative stress markers in US adults: A cross-sectional study

**DOI:** 10.18332/tid/216136

**Published:** 2026-02-06

**Authors:** Hang Zhong, Shifu Bao, Wanquan Cao, Xin He, Zhaonan Ban

**Affiliations:** 1 Department of Orthopedics, The Fifth People's Hospital Affiliated to Chengdu University of Traditional Chinese Medicine, Chengdu, China

**Keywords:** phenoage, smoke, serum cotinine, cross-sectional study, NHANES

## Abstract

**INTRODUCTION:**

Tobacco exposure is a plausible accelerator of biological aging, yet population-level evidence and mechanisms remain insufficiently defined. We examined the association between serum cotinine and phenotypic age acceleration (PhenoAgeAccel), and assessed whether oxidative-stress biomarkers were related to the serum cotinine–PhenoAgeAccel association.

**METHODS:**

We conducted a cross-sectional, survey-weighted analysis of n=19744 adults from NHANES 2011–2018. PhenoAgeAccel was computed as the residual from regressing PhenoAge on chronological age. Multivariable linear regressions related serum cotinine to PhenoAgeAccel across hierarchical adjustment models. Restricted cubic splines assessed non-linearity. Mediation analysis was conducted to quantify the extent to which oxidative-stress biomarkers contribute to this association.

**RESULTS:**

Higher serum cotinine was associated with accelerated biological aging: each doubling of serum cotinine corresponded to a 0.22-year increase in PhenoAgeAccel (β=0.22; 95% CI: 0.16–0.29). Mediation analyses indicated that γ-glutamyl transferase (GGT) and uric acid (UA) statistically accounted for 9.5% of the association between serum cotinine and PhenoAgeAccel (p<0.001). Interactions were observed for sex and PIR, with stronger associations among women and participants with lower socioeconomic status. There was no evidence of non-linearity in the relationships of the serum cotinine with GGT, PhenoAgeAccel, or UA.

**CONCLUSIONS:**

In this nationally representative cross-sectional study of US adults, higher serum cotinine levels were associated with greater phenotypic age acceleration. Oxidative-stress biomarkers were related to the observed association, although causal inferences cannot be drawn.

## INTRODUCTION

Aging is a universal biological process accompanied by progressive functional decline and heightened vulnerability to disease. By 2030, approximately one-sixth of the global population will be aged ≥60 years^1^. Aging is inherently multifactorial, reflecting the concerted dysfunction of multiple physiological systems^2^. It is marked by cumulative molecular perturbations and the disruption of hallmark processes, including genomic instability, telomere attrition, and stem-cell exhaustion^3^. Accelerated aging confers increased susceptibility to chronic disease and elevates mortality risk. Although chronological age remains the dominant correlate of aging-related outcomes, pronounced inter-individual heterogeneity persists among people of the same chronological age^4^. Identifying determinants of accelerated aging and developing interventions to slow, halt, or reverse these trajectories are essential to reducing disease burden and extending lifespan.

Phenotypic age (PhenoAge) is a machine-learning–derived measure of biological aging that integrates routine clinical biomarkers to improve estimation of biological age and enhance prediction of age-related disease risk^4,5^. PhenoAge also captures morbidity and mortality risk across diverse populations^6^ and has been widely deployed in studies of health risk and longevity^7,8^. Its acceleration metric, phenotypic age acceleration (PhenoAgeAccel) – the difference between PhenoAge and chronological age – quantifies deviation from expected aging; positive values indicate accelerated aging and are associated with higher health risks^5^. PhenoAgeAccel has proven informative in investigations of environment-linked aging, enabling the identification of high-risk subpopulations and guiding targeted health interventions^9^.

Cotinine, a long-lived nicotine metabolite, is widely regarded as a key biomarker for quantifying tobacco-smoke exposure^10^. Owing to its greater temporal stability in blood than in urine, serum cotinine is considered a more reliable indicator of exposure^11^. Beyond indexing exposure, cotinine levels correlate with adverse sequelae of smoking, including heightened oxidative stress and impairment of mesenchymal stem-cell function – processes implicated in morbidity and mortality.

While the cardiopulmonary harms of smoking are well recognized, its accelerating effects on the nervous system and cognitive aging merit equal attention^12^. Convergent evidence indicates that smoking can propel aging processes across multiple biological domains^13^. First, smoking exacerbates cellular aging through the induction of oxidative stress. High-dose nicotine and other tobacco constituents elicit oxidative damage consistent with core mechanisms of aging; antioxidant treatment can mitigate nicotine-induced spatial memory deficits, underscoring the central role of oxidative stress in this pathway^14,15^. Second, smoking perturbs endocrine homeostasis, notably the thyroid-hormone signaling axis. Because thyroid hormones are essential for adult cognitive function, disruption of this pathway provides a mechanistic rationale for earlier cognitive decline reported in smokers – and potentially in their offspring^16,17^.

Independent lines of evidence link aging with elevated oxidative stress^18^, which in turn accelerates telomere attrition^19^. A plausible underpinning is mitochondrial dysfunction – a hallmark contributor to aging – that increases the generation of reactive oxygen species while impairing endogenous antioxidant defences^20^. Consistent with this model, antioxidant-rich diets can attenuate cellular oxidative stress and may slow biological aging^21^.

Although tobacco exposure is associated with oxidative stress and age-related morbidity, its relationship with biological aging at the population level remains unclear. Serum cotinine is a robust marker of tobacco exposure and has been linked to oxidative stress, which is implicated in phenotypic age acceleration. However, few population-based studies have jointly evaluated serum cotinine, oxidative-stress biomarkers, and phenotypic aging, especially in nationally representative samples.

Guided by these observations, we examined whether oxidative-stress biomarkers were statistically related to the association between serum cotinine and PhenoAgeAccel. Accordingly, we used data from the National Health and Nutrition Examination Survey (NHANES) to examine the relationship between serum cotinine and PhenoAgeAccel, and to test mediation by oxidative-stress biomarkers, specifically serum γ-glutamyl transferase (GGT) and uric acid (UA).

## METHODS

**Figure 1:**
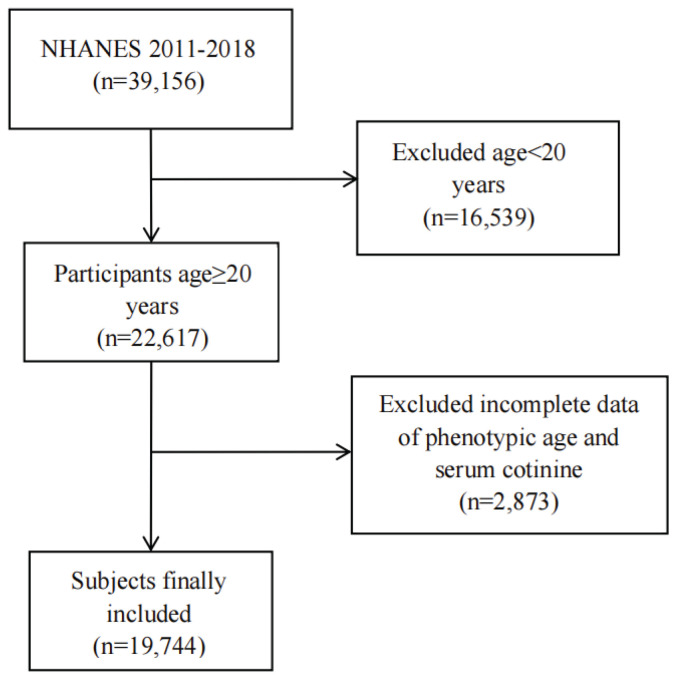
Flowchart for inclusion of participants, United States, NHANES 2011–2018 (N=19744)

This cross-sectional study draws on data from the NHANES, a nationally representative program that provides comprehensive information on the health and nutrition of the US population through standardized interviews and examinations^5^. The NHANES survey protocols were reviewed and approved by the NCHS Research Ethics Review Board (ERB). Specifically, the data used in this study were collected under Protocol #2011-17 (for 2011–2016 cycles) and Protocol #2018-01 (for 2017–2020 cycles). Participants were sampled from four NHANES cycles (2011–2018). Of 39156 individuals initially assessed, we excluded those younger than 20 years (n=16539), those missing PhenoAge data and cotinine data (n=2873), yielding a final analytical sample of 19744 adults aged ≥20 years. The screening flow is shown in Figure 1.

### Study variables

*Serum cotinine*

Venous blood was collected at mobile examination centers following NHANES standard operating procedures. Serum cotinine was quantified using isotope-dilution high-performance liquid chromatography with atmospheric-pressure chemical-ionization tandem mass spectrometry (ID HPLC–APCI–MS/MS).

*PhenoAge and PhenoAgeAccel*

PhenoAge was derived from nine clinical biomarkers – albumin, creatinine, fasting glucose, C-reactive protein (CRP), lymphocyte percentage, mean corpuscular volume, red cell distribution width, alkaline phosphatase and white blood cell count – together with chronological age^6^. We implemented the PhenoAge model trained on NHANES III using the *BioAge* R package and applied it to NHANES IV data (1999–2018). Because CRP was unavailable in NHANES 2011–2018, it was omitted; comparison of PhenoAge computed with versus without CRP using 1999–2010 data, demonstrated high concordance (correlation coefficient 0.959–0.996)^22^, indicating that exclusion of CRP does not materially affect the estimate.

PhenoAgeAccel was defined as the residual from a linear regression of PhenoAge on chronological age^8^. Participants with PhenoAgeAccel >0 were classified as phenotypically older, and those with PhenoAgeAccel <0 as phenotypically younger.

*Oxidative-stress biomarkers*

We considered GGT and UA as biomarkers linked to oxidative stress. Both were measured on a Beckman Coulter UniCel Dx800 analyser (Brea, CA, USA). GGT activity was assayed by an enzymatic-rate method, and UA by an endpoint method. Detailed laboratory protocols are available on the NHANES website.

*Covariates*

Referring to prior research and clinical insights, we accounted for covariates that could potentially impact the link between serum cotinine and Phenoage. The covariates in this study included age (years), gender (male, female), race (American, White, Black, other), marital status(married/living with a partner and widowed/divorced/separated/never married), education level (Lower than 12th grade, high school graduate or equivalent, some college or AA degree, and college graduate or higher), poverty income ratio (PIR), smoke(never, former and now), drinking status (never, former, mild, moderate, heavy), physical activity (yes, no), hypertension (yes, no), diabetes (yes, no), cardiovascular disease (yes, no), body mass index (BMI kg/m2), total protein(g/dL), blood urea nitrogen (mg/dL), serum creatinine (mg/dL), serum calcium (mg/dL), alkaline phosphatase (u/L), and serum phosphorus (mg/dL)^23^. Physical activity (PA) data were converted to metabolic equivalent minutes of moderate to vigorous physical activity per week (MET). Respondents were classified based on the criterion of meeting MET (≥600 MET-minutes/week, equivalent to 150 min/week of moderate-intensity or 75 min/week of vigorous-intensity physical activity) or not meeting the recommendation guidelines for adults (<600 MET-minutes/week)^24^.

### Statistical analysis

All analyses incorporated the NHANES complex survey design – sampling weights, strata, and primary sampling units – in accordance with guidance from the Centers for Disease Control and Prevention (CDC) and the National Center for Health Statistics (NCHS). For multi-cycle analyses, sampling weights were re-derived following NCHS analytic guidelines. Continuous variables are expressed as means with standard error (SE), while categorical variables are represented as frequencies (n) and proportions (%). Baseline characteristics were compared using survey-weighted χ^2^ tests for categorical variables and survey-weighted one-way ANOVA for continuous variables. The association between serum cotinine and PhenoAgeAccel was examined using multivariable linear regression under three specifications. Model 1 was unadjusted. Model 2 adjusted for age, gender, and race. Model 3 further adjusted for marital status, education level, PIR, smoking status, drinking status, physical activity, hypertension, diabetes, CVD, BMI, total protein, blood urea nitrogen, serum creatinine, serum calcium, alkaline phosphatase (ALP), and serum phosphorus. Serum cotinine was analyzed both as a continuous variable and as categorical tertiles. Because cotinine was right-skewed, values were log10-transformed; regression coefficients therefore represent the change in PhenoAgeAccel per doubling of serum cotinine.

Prespecified subgroup analyses stratified the association by age (≤45 vs >45 years), gender (female vs male), race (White vs Black vs Mexican American vs other), PIR (<1 vs 1–3 vs >3), physical activity (no vs yes), smoking status (never vs former vs current), BMI (<25 vs 25–30 vs ≥30 kg/m^2^), hypertension (yes/no) and diabetes (yes, no). These factors were treated as potential effect modifiers^25^.

To assess mediation by oxidative-stress biomarkers, we used the mediation R package (version 4.1) with 5000 non-parametric bootstrap resamples. We estimated the total effect of the serum cotinine on PhenoAgeAccel, the average direct effect and the average causal mediation effect through oxidative-stress biomarkers; the proportion mediated was calculated as the indirect effect divided by the total effect. Restricted cubic spline models were additionally applied to explore potential nonlinear associations between serum cotinine and PhenoAgeAccel. All data processing and statistical analyses were conducted in R 4.1.3. Statistical significance was defined as two-sided p<0.05.

## RESULTS

### Baseline characteristics of participants

As shown in Table 1, participants were grouped by tertiles of serum cotinine. A total of 19744 individuals were included (48.24% male, 51.76% female; mean age 47.75 ± 0.30 years). Across the serum cotinine tertiles, we observed significant differences in age, gender, race, PIR, BMI, education level, marital status, smoking and drinking status, PA, PhenoAge, serum uric acid, blood urea nitrogen, ALP, and serum creatinine (all p<0.05). Individuals with higher cotinine concentrations tended to be younger, male, White, married, current smokers, from lower income strata, and to have higher BMI, ALP, serum creatinine, and uric acid; they also exhibited higher education level and greater physical activity.

**Table 1:** Characteristics of the study participants according to serum cotinine levels, United States, NHANES 2011–2018 (N=19744)

*Variables*	*Total* *(N=19744)* *n (%)*	*T1* *(N=6865)* *n (%)*	*T2* *(N=6307)* *n (%)*	*T3* *(N=6572)* *n (%)*	*p*
**Age** (years), mean ± SE	47.75 ± 0.30	51.49 ± 0.44	47.48 ± 0.40	43.47 ± 0.39	<0.0001
**Gender**					<0.0001
Female	10201 (51.76)	4017 (57.98)	3412 (53.13)	2772 (42.98)	
Male	9543 (48.24)	2848 (42.02)	2895 (46.87)	3800 (57.02)	
**Race**					<0.0001
Black	4271 (10.62)	833 (5.25)	1366 (11.34)	2072 (16.44)	
Mexican American	2709 (8.68)	1313 (10.25)	818 (9.18)	578 (6.33)	
Other	5323 (15.22)	1931 (13.87)	2124 (19.40)	1268 (12.99)	
White	7441 (65.48)	2788 (70.63)	1999 (60.08)	2654 (64.24)	
**Education level**					<0.0001
Lower than 12th grade	4272 (14.05)	1269 (9.82)	1307 (13.53)	1696 (19.62)	
High school grade or equivalent	4386 (22.38)	1164 (15.97)	1324 (21.84)	1898 (30.61)	
Some college or AA degree	6114 (32.21)	1960 (28.75)	1920 (33.24)	2234 (35.43)	
College graduate or higher	4952 (31.31)	2467 (45.42)	1747 (31.30)	738 (14.29)	
**Marital status**					<0.0001
Divorced	2151 (10.22)	588 (8.32)	667 (9.85)	896 (12.84)	
Living with partner	1654 (8.45)	348 (4.47)	461 (8.44)	845 (13.25)	
Married	10039 (54.68)	4330 (67.35)	3327 (54.44)	2382 (39.60)	
Never married	3748 (18.53)	848 (11.86)	1141 (18.95)	1759 (26.18)	
Separated	669 (2.44)	151 (1.32)	209 (2.36)	309 (3.87)	
Widowed	1483 (5.67)	600 (6.66)	502 (5.92)	381 (4.24)	
**Smoking status**					<0.0001
Never	11332 (56.61)	5125 (73.85)	4526 (70.37)	1681 (23.20)	
Former	4609 (24.65)	1715 (25.81)	1726 (28.77)	1168 (19.50)	
Now	3789 (18.70)	21 (0.34)	49 (0.86)	3719 (57.29)	
**Drinking status**					<0.0001
Never	2542 (9.70)	1140 (13.85)	1014 (13.19)	388 (5.18)	
Former	2301 (9.76)	809 (10.67)	773 (11.52)	719 (10.86)	
Mild	6087 (33.94)	2475 (46.67)	1967 (39.07)	1645 (26.91)	
Moderate	2782 (16.40)	918 (18.11)	814 (17.89)	1050 (19.39)	
Heavy	3401 (19.10)	632 (10.70)	845 (18.32)	1924 (37.66)	
**Diabetes**					0.004
No	15793 (84.49)	18230 (83.23)	18436 (84.18)	18615 (86.31)	
Yes	3951 (15.51)	1514 (16.77)	1308 (15.82)	1129 (13.69)	
**Hypertension**					0.06
No	11295 (61.54)	3784 (60.09)	3653 (62.42)	3858 (62.47)	
Yes	8449 (38.46)	3081 (39.91)	2654 (37.58)	2714 (37.53)	
**Cardiovascular diseases**					0.11
No	17638 (91.15)	6149 (91.55)	5676 (91.71)	5811 (90.14)	
Yes	2106 (8.85)	716 (8.44)	631 (8.29)	761 (9.85)	
**Physical activity**					<0.001
no PA	2711 (13.02)	1035 (14.77)	910 (12.98)	766 (10.94)	
PA	17033 (86.99)	5830 (85.24)	5397 (87.02)	5806 (89.06)	
PIR, mean ± SE	2.98 ± 0.05	3.46 ± 0.05	3.00 ± 0.05	2.38 ± 0.05	<0.0001
	**Mean ± SE**	**Mean ± SE**	**Mean ± SE**	**Mean ± SE**	
BMI (kg/m²)	29.29 ± 0.11	29.12 ± 0.14	29.70 ± 0.18	29.12 ± 0.14	0.003
PhenoAge	45.42 ± 0.32	48.53 ± 0.46	44.74 ± 0.46	42.29 ± 0.40	<0.0001
PhenoAgeAccel	-2.33 ± 0.09	-2.96 ± 0.11	-2.74 ± 0.12	-1.19 ± 0.11	<0.0001
Alkaline phosphatase (U/L)	68.44 ± 0.32	67.26 ± 0.45	67.95 ± 0.43	70.32 ± 0.41	<0.0001
Serum creatinine (mg/dL)	0.87 ± 0.00	0.86 ± 0.00	0.87 ± 0.00	0.88 ± 0.00	0.003
Total protein (g/dL)	7.08 ± 0.01	7.05 ± 0.01	7.11 ± 0.01	7.10 ± 0.01	<0.0001
Serum uric acid (mg/dL)	5.39 ± 0.02	5.27 ± 0.02	5.45 ± 0.03	5.50 ± 0.03	< 0.0001
Blood urea nitrogen (mg/dL)	13.93 ± 0.09	14.74 ± 0.14	14.09 ± 0.10	12.80 ± 0.09	<0.0001
Serum phosphorus (mg/dL)	3.71 ± 0.01	3.70 ± 0.01	3.70 ± 0.01	3.72 ± 0.01	0.22
Serum calcium (mg/dL)	9.37 ± 0.01	9.37 ± 0.01	9.36 ± 0.01	9.38 ± 0.01	0.37
γ-glutamyl transferase (U/L)	27.43 ± 0.32	24.97 ± 0.56	25.98 ± 0.49	31.73 ± 0.63	<0.0001

BMI: body mass index. PIR: poverty income ratio. PhenoAge: phenotypic age. PhenoAgeAccel: phenotypic age acceleration. PA: physical activity. T1: cotinine level <0.016 ng/mL. T2: 0.016≤ cotinine level ≤0.185 ng/mL. T3: cotinine level ≥0.185 ng/mL. P-values were calculated using weighted chi-squared tests for categorical variables and one-way analysis of variance (ANOVA) for continuous variables.

### Association between serum cotinine and PhenoAgeAccel

Table 2 summarizes multivariable survey-weighted regressions relating cotinine to PhenoAgeAccel. Higher cotinine was associated with greater PhenoAgeAccel. In the fully adjusted model (Model 3), each doubling of cotinine was associated with a 0.22-year increase in PhenoAgeAccel (β=0.22; 95% CI: 0.16–0.29, p<0.0001). Treating cotinine as tertiles for sensitivity analysis yielded consistent results: compared with tertile 1, tertile 3 showed an adjusted β of 0.50 years (95% CI: 0.29–0.72, p<0.0001).

**Table 2:** Association between the serum cotinine and PhenoAge Acceleration (PhenoAgeAccel), multivariable linear regression, United States, NHANES 2011–2018 (N=19744)

*Serum cotinine (ng/mL)*	*Model 1* *β (95% CI) p*	*Model 2* *β (95% CI) p*	*Model 3* *β (95% CI) p*
Log10-transformed cotinine	0.5 (0.44–0.57) <0.0001	0.54 (0.47–0.60) <0.0001	0.22 (0.16–0.29) <0.0001
**Categories**			
T1 (ref.)			
T2	0.22 (-0.04–0.47) 0.10	0.31 (0.07–0.55) 0.01	-0.04 (-0.24–0.15) 0.65
T3	1.77 (1.52–2.02) <0.0001	1.94 (1.68–2.20) <0.0001	0.5 (0.29– 0.72) <0.0001
p for trend	<0.0001	<0.0001	<0.001

Model 1 was unadjusted. Model 2 was adjusted for gender, age, and race. Model 3 included additional adjustments for marital status, education level, poverty income ratio, smoking status, drinking status, physical activity, hypertension, diabetes, cardiovascular diseases, body mass index, and biochemical markers (total protein, blood urea nitrogen, serum creatinine, serum calcium, alkaline phosphatase, and serum phosphorus). T1: cotinine level <0.016 ng/mL. T2: 0.016≤ cotinine level ≤0.185 ng/mL. T3: cotinine level ≥0.185 ng/mL. Analyses incorporated NHANES survey sampling weights to account for the complex survey design.

### Association between serum cotinine and GGT

Table 3 presents associations of cotinine with γ-glutamyl transferase (GGT). In Model 3, each doubling of cotinine corresponded to a 1.52-U/L higher GGT (β=1.52; 95% CI: 1.16–2.21, p<0.001). Categorizing cotinine into tertiles produced similar inferences: the highest tertile exhibited a 6.24-U/L higher GGT relative to the lowest (β=6.24; 95% CI: 4.11–8.58, p<0.001).

**Table 3:** Association between the serum cotinine and γ-glutamyl transferase (GGT), serum cotinine and uric acid (UA), multivariable linear regression, United States, NHANES 2011–2018 (N=19744)

	*Model 1* *β (95% CI) p*	*Model 2* *β (95% CI) p*	*Model 3* *β (95% CI) p*
**Serum cotinine (ng/mL) and γ-glutamyl transferase**			
Log10-transformed cotinine	1.78 (1.31–2.25) <0.0001	1.72 ( 1.27–2.18) <0.0001	1.52 (1.16–2.21) <0.001
**Categories**			
T1 (ref.)			
T2	1.01 (-0.40–2.42) 0.16	0.9 (-0.57–2.38) 0.23	-0.33 (-1.88–1.22) 0.66
T3	6.76 (4.95–8.57) <0.0001	6.59 (4.78–8.39) <0.0001	6.24 (4.11–8.58) <0.001
p for trend	<0.0001	<0.0001	0.17
**Serum cotinine (ng/mL) and uric acid**			
Log10-transformed cotinine	0.03 (0.02–0.05) <0.001	-0.01 (-0.02–0.01) 0.44	0.02 (0.01–0.05) 0.007
**Categories**			
T1 (ref.)			
T2	0.18 (0.10–0.26) <0.0001	0.15 (0.09–0.21) <0.0001	0.08 (0.03–0.14) 0.01
T3	0.23 (0.16–0.31) <0.0001	0.1 (0.04–0.16) 0.003	0.14 (0.05–0.22) 0.003
p for trend	0.11	0.09	0.74

Model 1 was unadjusted. Model 2 was adjusted for gender, age, and race. Model 3 included additional adjustments for marital status, education level, poverty income ratio, smoking status, drinking status, physical activity, hypertension, diabetes, cardiovascular diseases, body mass index, and biochemical markers (total protein, blood urea nitrogen, serum creatinine, serum calcium, alkaline phosphatase, and serum phosphorus). T1: cotinine level <0.016 ng/mL. T2: 0.016≤ cotinine level ≤0.185 ng/mL. T3: cotinine level ≥0.185 ng/mL. Analyses incorporated NHANES survey sampling weights to account for the complex survey design.

### Association between serum cotinine and UA

Table 3 also shows associations of cotinine with serum uric acid. In Model 3, each doubling of cotinine was associated with a 0.02 mg/dL higher uric acid (β=0.02; 95% CI: 0.01–0.05, p=0.007). In tertile analyses, the highest versus lowest cotinine tertile was associated with a 0.14 mg/dL higher uric acid (β=0.14; 95% CI: 0.05–0.22, p<0.001).

### Associations of GGT and UA with PhenoAgeAccel

As reported in Table 4, both biomarkers were positively related to PhenoAgeAccel. For GGT, each 1-U/L increment corresponded to a 0.01-year higher PhenoAgeAccel (β=0.01; 95% CI: 0.01–0.02, p<0.001). In quartile analyses, participants in the highest GGT quartile (Q4) had significantly higher PhenoAgeAccel compared with those in the lowest quartile (Q1) (β=0.28; 95% CI: 0.07–0.49, p=0.01), whereas no statistically significant differences were observed for the second (Q2) or third (Q3) quartiles. For uric acid, each 1 mg/dL increment corresponded to a 0.38-year higher PhenoAgeAccel (β=0.38; 95% CI: 0.31–0.45, p<0.0001). In quartile-based analyses, compared with participants in the lowest quartile (Q1), those in the highest quartile (Q4) had substantially higher PhenoAgeAccel (β=1.10; 95% CI: 0.83–1.37, p<0.001), whereas smaller effect sizes were observed for the second (Q2) and third (Q3) quartiles. A significant dose–response trend across quartiles was observed (p for trend <0.001).

**Table 4:** Association between the uric acid (UA), γ-glutamyl transferase (GGT) and PhenoAge Acceleration (PhenoAgeAccel), multivariable linear regression, United States, NHANES 2011–2018 (N=19744)

	*Model 1* *β (95% CI) p*	*Model 2* *β (95% CI) p*	*Model 3* *β (95% CI) p*
**Uric acid** (mg/dL)			
**Continuous** (per mg/dL)	0.68 (0.61–0.75) <0.0001	0.63 (0.56–0.70) <0.0001	0.38 (0.31–0.45) <0.0001
**Categories**			
Q1 (ref.)			
Q2	0.58 (0.33–0.83) <0.0001	0.48 (0.23–0.74) <0.001	0.23 (0.03–0.43) 0.03
Q3	0.95 (0.68–1.22) <0.0001	0.74 (0.46–1.02) <0.0001	0.26 (0.01–0.52) 0.05
Q4	2.33 (2.06–2.60) <0.0001	2.05 (1.77–2.32) <0.0001	1.1 (0.83–1.37) <0.0001
p for trend	<0.0001	<0.0001	<0.0001
**GGT** (U/L)			
**Continuous** (per U/L)	0.02 (001–0.02) <0.0001	0.01 (0.0–0.02) <0.0001	0.01 (0.01–0.02) <0.001
**Categories**			
Q1 (ref.)			
Q2	0.72 (0.50–0.95) <0.0001	0.48 (0.26–0.70) <0.0001	0 (-0.18–0.19) 1.00
Q3	1.43 (1.18–1.68) <0.0001	1.08 (0.81–1.34) <0.0001	0.14 (-0.06–0.34) 0.15
Q4	2.23 (1.98–2.47) <0.0001	1.86 (1.61–2.10) <0.0001	0.28 (0.07–0.49) 0.01
p for trend	<0.0001	<0.0001	0.01

Model 1 was unadjusted. Model 2 was adjusted for gender, age, and race. Model 3 included additional adjustments for marital status, education level, poverty income ratio, smoking status, drinking status, physical activity, hypertension, diabetes, cardiovascular diseases, body mass index, and biochemical markers (total protein, blood urea nitrogen, serum creatinine, serum calcium, alkaline phosphatase, and serum phosphorus). T1: cotinine level <0.016 ng/mL. T2: 0.016≤ cotinine level ≤0.185 ng/mL. T3: cotinine level ≥0.185 ng/mL. Analyses incorporated NHANES survey sampling weights to account for the complex survey design. Q1–Q4: quartiles.

### Mediation analyses

**Figure 2:**
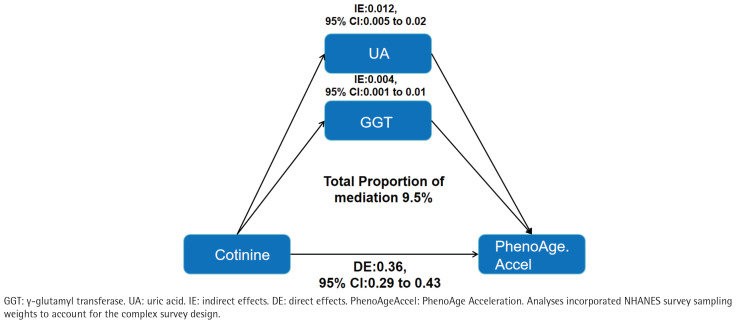
Indirect effects of oxidative-stress biomarkers in the association between serum cotinine and PhenoAgeAccel , United States, NHANES 2011–2018 (N=19744)

Parallel mediation analyses evaluated oxidative-stress pathways. The direct effect of serum cotinine on PhenoAgeAccel remained statistically significant (direct effect =0.36; 95% CI: 0.29–0.43). Individually, both GGT and uric acid showed significant mediation of the cotinine–PhenoAgeAccel association, with mediation proportions of 3.5% and 6.0%, respectively (both p<0.05). In a multivariable model including both mediators, the combined indirect effect remained significant, accounting for 9.5% of the total effect (Figure 2).

### Subgroup analyses

**Figure 3:**
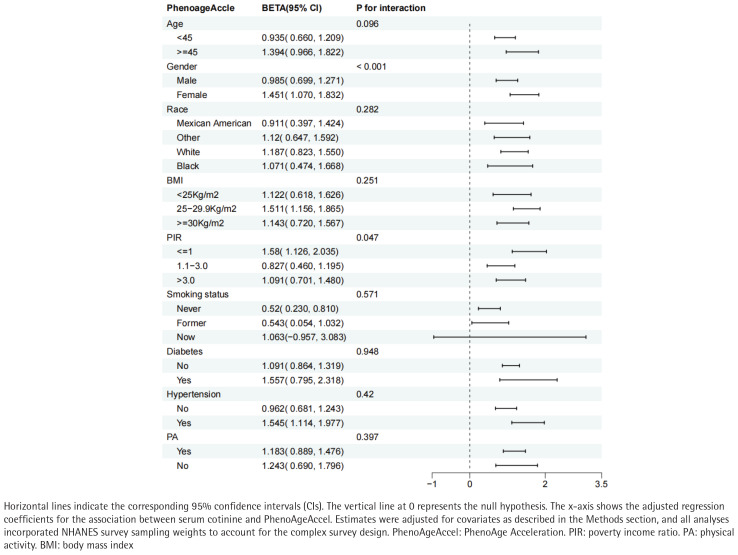
Subgroup analysis for the association between serum cotinine and PhenoAgeAccel, United States, NHANES 2011–2018 (N=19744)

We detected significant effect modification by sex (interaction p<0.001) and PIR (interaction p=0.047). The cotinine–PhenoAgeAccel association was more pronounced among women and among participants with lower socioeconomic status. (Figure 3).

### Restricted cubic splines

**Figure 4:**
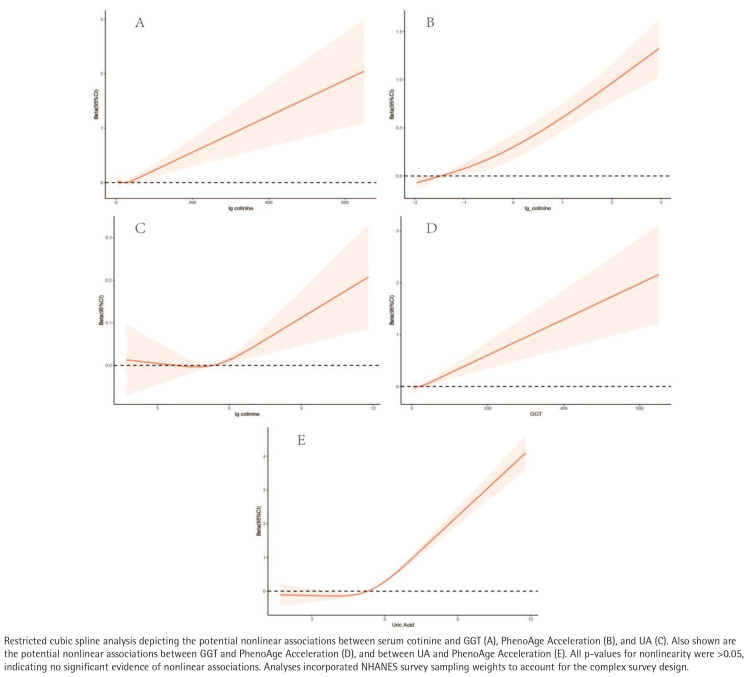
Restricted cubic spline analyses of serum cotinine, γ-glutamyl transferase (GGT), uric acid (UA), and PhenoAge Acceleration, United States, NHANES 2011–2018 (N=19744)

After multivariable adjustment, there was no evidence of non-linearity in the relationships of the serum cotinine with GGT, PhenoAgeAccel or UA (all *p* for non-linearity >0.05). Each outcome increased monotonically with higher serum cotinine (Figure 4).

## DISCUSSION

Our novel study examined the association between serum cotinine and PhenoAgeAccel in a nationally representative US population. We observed a robust positive association: in fully adjusted models, each doubling of serum cotinine corresponded to a 0.22-year increase in PhenoAgeAccel. Mediation analyses further supported a contributory role for oxidative stress, with GGT and UA jointly mediating part of the serum cotinine–PhenoAgeAccel association. Subgroup analyses further indicated stronger associations among women and participants with lower socioeconomic status. This heterogeneity may reflect differences in vulnerability, exposure patterns, or unmeasured social and behavioral factors, warranting further investigation. These findings contribute to understanding the population-level associations between tobacco exposure and phenotypic aging.

Cotinine is the biomarker of choice for tobacco exposure assessment owing to its higher concentrations and longer elimination half-life relative to nicotine^11,26^. Smoking accelerates aging through convergent molecular and cellular pathways with systemic and persistent effects. First, smoking provokes chronic inflammation and immune activation, yielding molecular features that recapitulate aspects of normative aging. A study reported hypomethylation and upregulation of immune-related regulatory elements alongside hypermethylation at Polycomb repressive complex (PRC) targets, perturbing epigenetic homeostasis and promoting sustained inflammation and tissue damage^13^. Second, tobacco toxins activate the aryl-hydrocarbon-receptor (AHR) axis (including AHRR, *CYP1A1* and *CYP1B1*), escalating oxidative stress and DNA damage; the cumulative changes trigger DNA-repair–linked methylation shifts and advance epigenetic age^12,27^. In smokers, epigenetic age in airway and lung tissues is accelerated by 4–5 years, and lifelong exposure is associated with higher GrimAge and PhenoAge estimates and increased risks of cancer, cardiovascular and pulmonary disease^28^. At the vascular interface, prior work indicates that tobacco smoke suppresses collagen synthesis, induces matrix metalloproteinases and damages elastic fibers; reactive oxygen species (ROS) accumulation further accelerates extracellular-matrix degradation^28^. Collectively, smoking reshapes the tissue microenvironment and hastens aging via amplification of inflammation, oxidative stress, DNA-damage/repair imbalance and epigenetic drift.

Oxidative stress is a hallmark correlate of aging and is linked to telomere attrition and vulnerability to age-related disease^29^. It also impairs mitochondrial function, increasing ROS generation, mitochondrial dysfunction is a central driver of aging biology^30^. Our results align with prior evidence that higher PhenoAgeAccel tracks with GGT and UA levels^31^. Emerging data suggest that improving cardiovascular risk profiles – healthy diet, smoking cessation, adequate sleep, and control of BMI, glycaemia and blood pressure – can lower systemic oxidative stress^32,33^, underscoring the modifiability of these pathways.

To date, no epidemiological evidence suggests that cigarette smoking slows biological aging. A small body of preclinical research has reported potential anti-aging effects of low-dose nicotine in animal models, primarily through metabolic regulation and neuroprotective pathways^34^. However, these findings differ fundamentally from population-based studies of cigarette smoking, as they typically involve isolated nicotine administration under controlled conditions and do not account for the complex mixture of toxicants present in tobacco smoke. Moreover, evidence derived from animal experiments or short-term interventions cannot be directly extrapolated to long-term human exposure. Differences in exposure source (nicotine vs cigarette smoke), dosage, duration, and outcome definitions (molecular or functional aging markers vs composite biological aging indices) may therefore explain the discrepancies between these experimental findings and the present population-level results. In addition, large-scale epidemiological studies consistently report accelerated epigenetic aging, increased morbidity, and reduced healthspan among smokers, further supporting the population-level relevance of our findings.

### Strengths and limitations

Strengths include the nationally representative design, large sample size and integration of oxidative-stress biomarkers with formal mediation analysis. We show that serum cotinine is positively associated with both GGT and UA and that these biomarkers partially mediate the serum cotinine–PhenoAge relationship, offering mechanistic insight. Limitations merit consideration. First, PhenoAgeAccel, while validated, is not the sole index of biological aging; complementary measures warrant evaluation. Second, our oxidative-stress panel was restricted to GGT and UA and did not incorporate thresholds or additional markers that distinguish oxidative-stress subtypes (e.g. acute vs chronic). Broader biomarker panels (e.g. lipid peroxidation products, antioxidant capacity, mitochondrial function assays) could refine phenotyping. Third, the cross-sectional design limits causal interpretation of the observed associations and precludes establishing temporal ordering between exposure, oxidative-stress biomarkers, and phenotypic age acceleration. Accordingly, the mediation analyses should be regarded as hypothesis-generating rather than confirmatory. Fourth, participants with missing data were excluded from the analysis. If these data were not missing completely at random, selection bias may have occurred. In addition, serum cotinine reflects recent tobacco exposure and may misclassify occasional or intermittent smokers, particularly in cross-sectional settings, potentially leading to exposure misclassification. Finally, despite extensive covariate adjustment, residual confounding by unmeasured factors cannot be excluded. These include dietary patterns and antioxidant intake (e.g. fruit and vegetable consumption)^35^ and sleep patterns^36^.

## CONCLUSIONS

Among US adults, higher serum cotinine levels were associated with greater phenotypic age acceleration, as indexed by PhenoAgeAccel. Mediation analyses suggested that oxidative-stress biomarkers were statistically related to this association, indicating a potential mechanistic link between tobacco exposure and biological aging processes. These findings provide population-level evidence describing the relationships among tobacco exposure, oxidative stress, and phenotypic aging, while longitudinal studies are needed to clarify temporality and underlying mechanisms.

## Data Availability

The datasets used in this study are available in online repositories. Details, including the repository names and accession numbers, can be accessed here: https://www.cdc.gov/nchs/nhanes/Default.htm
